# Overexpression of serine racemase in retina and overproduction of D-serine in eyes of streptozotocin-induced diabetic retinopathy

**DOI:** 10.1186/1742-2094-8-119

**Published:** 2011-09-22

**Authors:** Haiyan Jiang, Junxu Fang, Bo Wu, Guibin Yin, Lin Sun, Jia Qu, Steven W Barger, Shengzhou Wu

**Affiliations:** 1 School of Optometry and Ophthalmology and Eye Hospital, Wenzhou Medical College. 270 Xueyuan Road, Wenzhou, Zhejiang, 325003, P.R.China; 2 State Key Laboratory Cultivation Base and Key Laboratory of Vision Science, Ministry of Health, P.R.China and Zhejiang Provincial Key Laboratory of Ophthalmology and Optometry. 270 Xueyuan Road, Wenzhou, Zhejiang, 325003, P.R.China; 3 Laboratory Animal Center, Wenzhou Medical College, 325035, Zhejiang, P.R.China; 4 Department of Geriatrics, University of Arkansas for Medical Sciences, Little Rock, AR,72205, USA; 5 Geriatric Research Education and Clinical Center, Central Arkansas Veterans Healthcare System, Little Rock AR, 72205, USA

**Keywords:** diabetic retinopathy, inflammation, retinal ganglion cell, inner nuclear layer, glutamate

## Abstract

**Background:**

Recent data indicate that inflammatory mechanisms contribute to diabetic retinopathy (DR). We have determined that serine racemase (SR) expression is increased by inflammatory stimuli including liposaccharide (LPS), amyloid β-peptide (A-beta), and secreted amyloid precursor protein (sAPP); expression is decreased by the anti-inflammatory drug, dexamethasone. We tested possibility that SR and its product, D-serine, were altered in a rat model of DR.

**Methods:**

Intraperitoneal injection of streptozotocin (STZ; 70 mg/kg body weight) to Sprague-Dawley rats produced type-I diabetic mellitus (fasting blood sugar higher than 300 mg/dL). At 3 and 5 months after STZ or saline injection, retinas from some rats were subjected to cryosectioning for immunofluorescent analysis of SR and TUNEL assay of apoptosis. Retinal homogenates were used to detect SR levels and Jun N-terminal kinase (JNK) activation by immunoblotting. Aqueous humor and retina were also collected to assay for neurotransmitters, including glutamate and D-serine, by reverse-phase HPLC.

**Results:**

Compared to saline-injected rats, STZ-injected (diabetic) rats showed elevation of SR protein levels in retinal homogenates, attributed to the inner nuclear layer (INL) by immunofluorescence. Aqueous humor fluid from STZ-injected rats contained significantly higher levels of glutamate and D-serine compared to controls; by contrast, D-serine levels in retinas did not differ. Levels of activated JNK were elevated in diabetic retinas compared to controls.

**Conclusions:**

Increased expression of SR in retina and higher levels of glutamate and D-serine in aqueous humor of STZ-treated rats may result from activation of the JNK pathway in diabetic sequelae. Our data suggest that the inflammatory conditions that prevail during DR result in elevation of D-serine, a neurotransmitter contributing to glutamate toxicity, potentially exacerbating the death of retinal ganglion cells in this condition.

## Background

Diabetic retinopathy (DR) is a sight-threatening complication of diabetic mellitus that becomes prevalent after about a decade with disease. The natural history of DR has been divided into an early, nonproliferative stage, and a later, proliferative stage. Multiple etiologic hypotheses have been proposed, including protein kinase C activation [1,2], excessive production of advanced glycation end products (AGEs) [3,4], and reactive oxygen species stemming from overconsumption of NAPDH as a result of overactivation of aldose reductase activity [5-7]. The pathology of DR involves microvasular changes, including blood-retinal barrier (BRB) breakdown, microaneurysm, increased expression of intercellular adhesion molecule 1 (ICAM-1), and death of endothelial cells and pericytes [8-11]. These microvascular changes frequently accompany inflammation. In addition to inflammation-related changes in retinal vessels, DR also involves neurodegeneration in the retinal ganglion cell layer (RGCL) and inner nuclear layer (INL) [12]; some evidence indicates this neuronal cell death precedes vascular changes in DR [12,13]. Excitotoxins including homocysteine and glutamate can induce toxicity in RGCs [14]; increased retinal glutamate is also found in the streptozotocin (STZ)-induced model of diabetes [15]. Recently, excitotoxicity contributing to neural degeneration was also linked to activity of serine racemase (SR), an enzyme that converts L-serine to its dextrarotatory enantiomer [16-19]. Whole-cell recording in rat retinas has indicated that D-serine enhances currents transmitted by N-methyl D-aspartate (NMDA) receptors, and removal of D-serine by D-amino acid oxidase (DAAOx) returned the currents to control amplitudes [20].

SR has been widely studied in recent decades. In neural tissues, it was initially identified in protoplasmic astrocytes [21], then microglia [16], and later in Schwann cells [22]. Its product D-serine acts as an agonist at the glycine_B _site of the NMDA receptor and influences neurotransmission [20]. Shortages of D-serine in the CNS have been linked to schizophrenia [23]. D-serine administration has helped to reverse negative symptoms of schizophrenia in clinical trials of combinatorial treatment regimens [24], and a loss-of-function mutation in SR produces schizophrenia-related behaviors in mice [25]. Overproduction of D-serine has been associated with excitotoxicity *in vitro *[16], amyotrophic lateral sclerosis [26], and experimental epilepsy [27]. Targeted knockout of serine racemase protects against toxicity of amyloid β-peptide (Aβ) and ischemic injury [18,19].

Regulation of serine racemase occurs at transcriptional, translational, and post-translational levels. Phosphorylation of SR at Thr-71 increases SR activity [28], and inhibition of proteasome activity increases SR protein levels [29]. At the transcriptional level, inflammatory stimuli--including Aβ, lipopolysaccharide (LPS) [16], and secreted amyloid precursor protein (sAPP)--increase SR mRNA [30]; and dexamethasone decreases SR mRNA [31]. Taken together, these lines of evidence suggest that inflammation regulates SR expression and thereby contributes to the etiology of DR. Therefore, we sought to determine whether production of SR and its product, D-serine, change in a model of DR utilizing the STZ-induced rat model of diabetes.

## Methods

### Materials

STZ was purchased from Sigma (St Louis, MO). Microsyringes and SR antibody were purchased from BD Biosciences (San Jose, CA). JNK, phospho-SAPK/JNK, phospho-c-Jun (Ser73), and GAPDH antibodies were purchased from Cell Signaling Technology, Inc. (Danvers, MA). An antibody detecting von Willebrand Factor (vWF) was purchased from Abcam (Cambridge, MA). Glucometer, *in situ *cell death detection kits, and fluorescein were purchased from Roche Diagnostics (Germany). Hematoxylin and eosin (H&E) were purchased from Beyotime Institute of Biotechnology (Beijing, China). CL-Xposure films were purchased from Thermo Scientific Branch (Shanghai, China). Pierce ECL Western Blotting Substrate was purchased from Thermo Scientific (Rockford, IL). Protease inhibitor cocktail was purchased from Calbiochem (San Diego, CA). Chloral hydrate, alcohol, and neutral balsam were purchased from Shanghai Pharmacy Company (Shanghai, China).

### Animals

Sprague-Dawley rats were purchased from the Shanghai Animal Experimental Center, Chinese Academy of Sciences and housed in standard pathogen-free (SPF) animal facilities with automatic illumination on a 12-h cycle at Wenzhou Medical College. All experiments were approved by the Wenzhou Medical College Committee according to Association for Research in Vision and Ophthalmology (ARVO) regulations on the use and care of animals.

### Establishment of DR rat model

Rats at 2 months of age were randomly assigned to groups receiving an intraperitoneal (i.p.) saline injection (N = 15) or a single i.p. injection of STZ (70 mg/kg body weight; N = 25). At the time of injection, the body weights within a given experimental group varied (249-281 g), but the mean body weights were identical for the STZ and saline groups. Blood glucose levels were monitored with a glucometer once a week, and final measurements were recorded at the end of the experiment immediately prior to euthanasia. Rats exhibiting fasting glucose levels in excess of 300 mg/dL were designated diabetic rats; STZ-injected rats not reaching this criterion were excluded from the experiments.

### Collection of aqueous humor and retinas

After anethesitizing rats with 10% chloral hydrate at 0.3 mL/100 g body weight, a microsyringe (300 μl) was inserted at the edge of cornea, and 20 μl of aqueous fluid was drawn from each eye. The rats were then euthanized, and the retinas were collected for analysis by immunoblotting or histology. Eyes were removed and opened by circumferential incision just below the ora serrata, and anterior segment and the vitreous were discarded. Under a dissection microscope, the retina was gently lifted off the eyecup.

### H&E staining

Retinas were immersion-fixed in 4% formaldehyde, dehydrated through graded ethanol steps and xylene, then embedded in paraffin. Sections were cut with a vibrotome (Leica RM 2135) at a thickness of 5 μm and mounted onto glass slides. The mounted sections were deparaffinized with xylene and rehydrated with graded ethanol steps from 100% to 70%. Hematoxylin was used to stain the sections for 3 min, followed by washing with tap water. After treatment with 0.1% HCl and 0.1% NH_4_OH, sections were exposed to eosin for 3 min, then dehydrated with graded ethanol steps and xylene, and coverslipped in neutral balsam. Observations were made under phase-contrast and bright-field microscopy (Olympus BX 41).

### TUNEL staining

Apoptosis was analyzed with the *In Situ *Cell Death Detection Kit (Roche). Frozen sections of the rat retinas were cut on a cryostat. The sections were postfixed with 4% paraformaldehyde and permeablized with 0.1% Triton X-100. A 50- μl TUNEL reaction mixture was added to each sample, and the slides were incubated in a humidified atmosphere for 60 min at 37°C in the dark and analyzed by fluorescence microscopy with an FITC filter.

### Western blotting for rat retinal homogenates

Retinas were homogenized with protein lysis buffer containing protease inhibitor cocktail and then centrifuged at 13,000 × g at 4°C for 10 min to remove insoluable pellets. The supernatants were quantified with BCA reagents (Beyotime Biotechnology). Retinal proteins (50 *g) from control or STZ-injected rats were loaded in individual lanes, resolved with SDS-PAGE analysis (12%), and then electrophoretically transferred to a nitrocellulose membrane. The transfer efficiency was monitored with Ponceau S (Sigma), and blots were blocked with 3% BSA or skim milk. SR antibody (1:500) or JNK/phospho-JNK antibody (1:1000) was diluted in Tris-buffered saline (pH 7.4) with 0.1% Tween-20 supplement (TBS-T) and applied to the blots overnight at 4°C. Following washes with TBS, a peroxidase-conjugated secondary antibody was applied at a dilution of 1:5000. Washes were followed by development with Pierce ECL Western Blotting Substrate. Each membrane probed for SR or JNK was stripped and probed for GAPDH detection.

### Immunofluorescence

Frozen sections of retina were blocked with skimmed milk overnight. SR antibody (1:100) in PBS containing 0.1% Triton X-100 was applied to the sections for 1 h at room temperature then overnight at 4°C. On the following day, the samples were washed three times with PBS and incubated for 1 h at room temperature with a secondary antibody conjugated to Alex Fluor 488 (1:1000). Following incubation in secondary antibody, the sections were washed in PBS at 4°C, coverslipped, and examined with a Zeiss Axiovert 200 equipped with epifluorescence optics. Images were recorded with a digital camera. Specificity was confirmed by omission of primary antibody.

### HPLC measurement of D-serine

Detection of D-serine by reverse-phase HPLC was performed using methods similar to those of Hashimoto *et al *[32]. Vitreous humor or retinas were collected as described above. Vitreous fluid or retinal homogenates were precipitated with 10% trichloroacetic acid (TCA) and cleared by centrifugation. TCA was removed from the supernatants with water-saturated ether, and they were then derivatized with a 3:7 mixture of solution A (30 mg/ml t-BOC-L-cysteine, 30 mg/ml *o*-phthaldialdehyde in methanol): solution B (100 mM sodium tetraborate solution, pH 9.4). A 3.5- μZORBAX Eclipse AAA column (150 × 4.6 mm) was used to separate the amino acids. A linear gradient was established from 100% buffer A (0.1 M sodium acetate buffer, pH 6; 7% acetonitrile; 3% tetrahydrofuran) to 100% buffer B (0.1 M sodium acetate buffer, pH 6; 4% acetonitrile; 3% tetrahydrofuran) over 60 min at 0.8 ml/min. Fluorescence was monitored with 344 nM excitation and 443 nM emission. In addition to their consistent retention times, D-serine peaks were confirmed by sensitivity to D-amino acid oxidase (DAAOx) digestion.

### Statistics

Pairwise comparisons between diabetic and control rats were assessed using Student's *t-test*. P ≤ 0.05 was accepted as indicative of a significant difference.

## Results

### Establishment of DR rat model

To examine the metabolic status of DR rats, we monitored fasting blood glucose once per week and body weights (BW) before and after STZ injection. The parameters for these experimental rats are summarized in Table 1.

**Table 1:** Weight change and fasting blood sugar of AMC and diabetic rats

Ages of rats (months) (months after manipulation)	Weight (g) Mean ± SEM	Fasting Blood Sugar (mg/dL) Mean ± SEM
2 (0, no treatment)	264.13 ± 4.26	105.98 ± 2.67

5 (3 mo. after saline)	599.25 ± 13.00	102.17 ± 2.79

5 (3 mo. after STZ)	222.13 ± 16.7 *	451.13 ± 11.61 *

7 (5 mo. after saline)	752.50 ± 26.58	103.05 ± 4.49

7 (5 mo. after STZ)	247.80 ± 5.25 *	460.44 + 18.73 *

* P < 0.05 compared to saline controls

**Figure 1:**
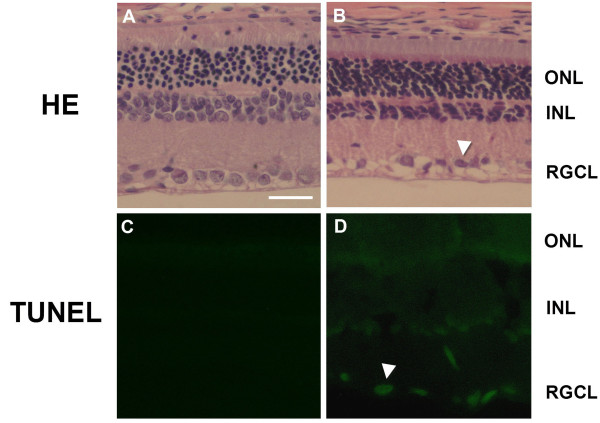
**Cellular death in retinas of DR rats**. The top images depict hematoxylin and eosin-stained cryosections of retinas of control (**A**) and DR rats (**B**) 3 months after onset of diabetes. The cells of the GCL are uniformly distributed in the control rats, whereas there is shrinkage and cell death occurring in the GCL (arrowhead) in DR rats. The bottom images show TUNEL staining of retinas of control (**C**) and DR rats (**D**) 3 months after onset of diabetes. DNA damage was apparent in the GCL and INL in the DR rats (arrow) but not in the AMC. RGCL, retinal ganglionic cell layer; INL, inner nuclear layer; ONL, outer nuclear layer. Scale bar = 50 μ.

A previous study demonstrated RGC loss occurs in DR model [33]. We examined RGCL integrity in our rat subjects with H&E and TUNEL staining. H&E staining indicated a reduction in the number of RGCs in some areas of RGCL in diabetic rats 3 months after STZ injection, as compared to the saline-injected group (Figure 1, A vs. 1B); similar effects were observed at 5 months after STZ injection (not shown). The INL in the diabetic group was thinner than that in the saline-injected group (Figure 1B). Positive TUNEL staining was found localized to the RGCL and INL in retinas of DR rats (Figure 1D), whereas no staining was detected in retinas of saline controls (Figure 1C).

### Increased SR expression in retinas of STZ-induced DR model

**Figure 2:**
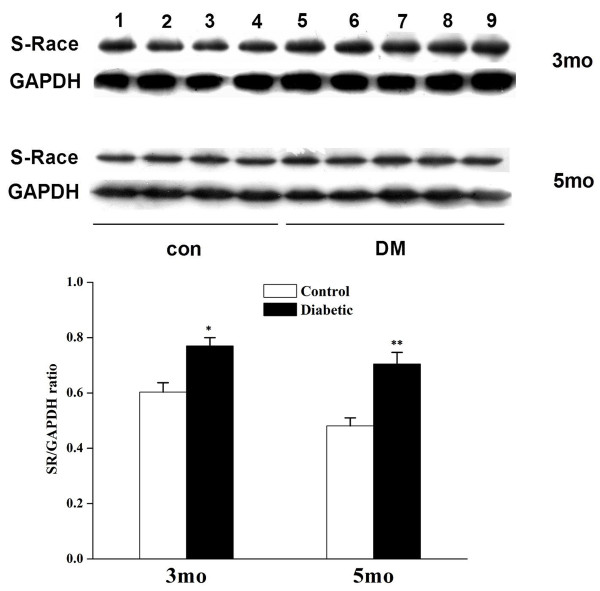
**Increased SR expression in retinas of DR rats**. **A: **Retinal homogenates from control and DR rats, 3 or 5 months after onset of diabetes, were subjected to immunoblotting for SR, with 50 μg total protein loaded in each lane. The left four lanes represent retinas of four control rats, whereas the right five lanes represent five DR rats. **B: **Densitometric scans indicate that the ratio between SR and GAPDH in DR rats is significantly higher than control (*P < 0.05 or **P < 0.05, DR vs. control; N: 8 control, 10 DR for each time point).

**Figure 3:**
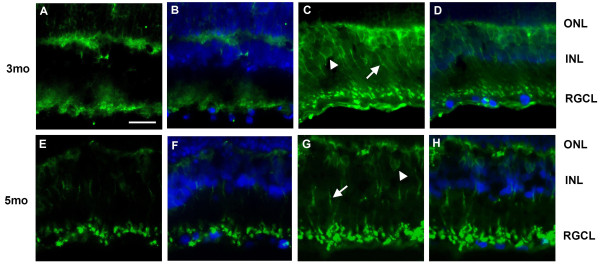
**Increased SR immunofluorescence in INL for retinas of DR rats**. **I**mmunofluorescence with SR was performed on cryosections from retinas of control (**A, E**) and DR rats (**C, G**) according to the procedures described in Methods. The SR immunofluorescence was merged with the DAPI staining (**B**,**D**; **F**,**H**), and increased staining was found to be predominantly in the INL (arrow and arrowhead, **C **and **G**) compared to the counterparts in control retinas (**A **and **E**). Green indicates SR staining and DAPI staining is blue. RGCL, retinal ganglionic cell layer; INL, inner nuclear layer; ONL, outer nuclear layer. Scale bar = 50 μ.

Previous studies have indicated that RGC death in DR may be associated with excitotoxicity [14,34]. Recent reports have indicated that D-serine can contribute to excitotoxicity [16-19,26]. Therefore, we tested whether SR or its product D-serine increases in eyes during STZ-induced DR. Retinas from DR and control rats were analyzed for SR expression, which was increased in DR compared to controls at 3 and 5 months post-STZ injection (Figure 2). To determine whether this increased expression may be attributable to the retinal layer, immunofluorescence was performed on cryosections. The results indicate that the increased staining was localized mostly in the INL at 3 and 5 months post-STZ injection (Figure 3C, G) compared to controls (Figure 3A, E).

### Increased D-serine and glutamate in aqueous humor of DR rats

**Figure 4:**
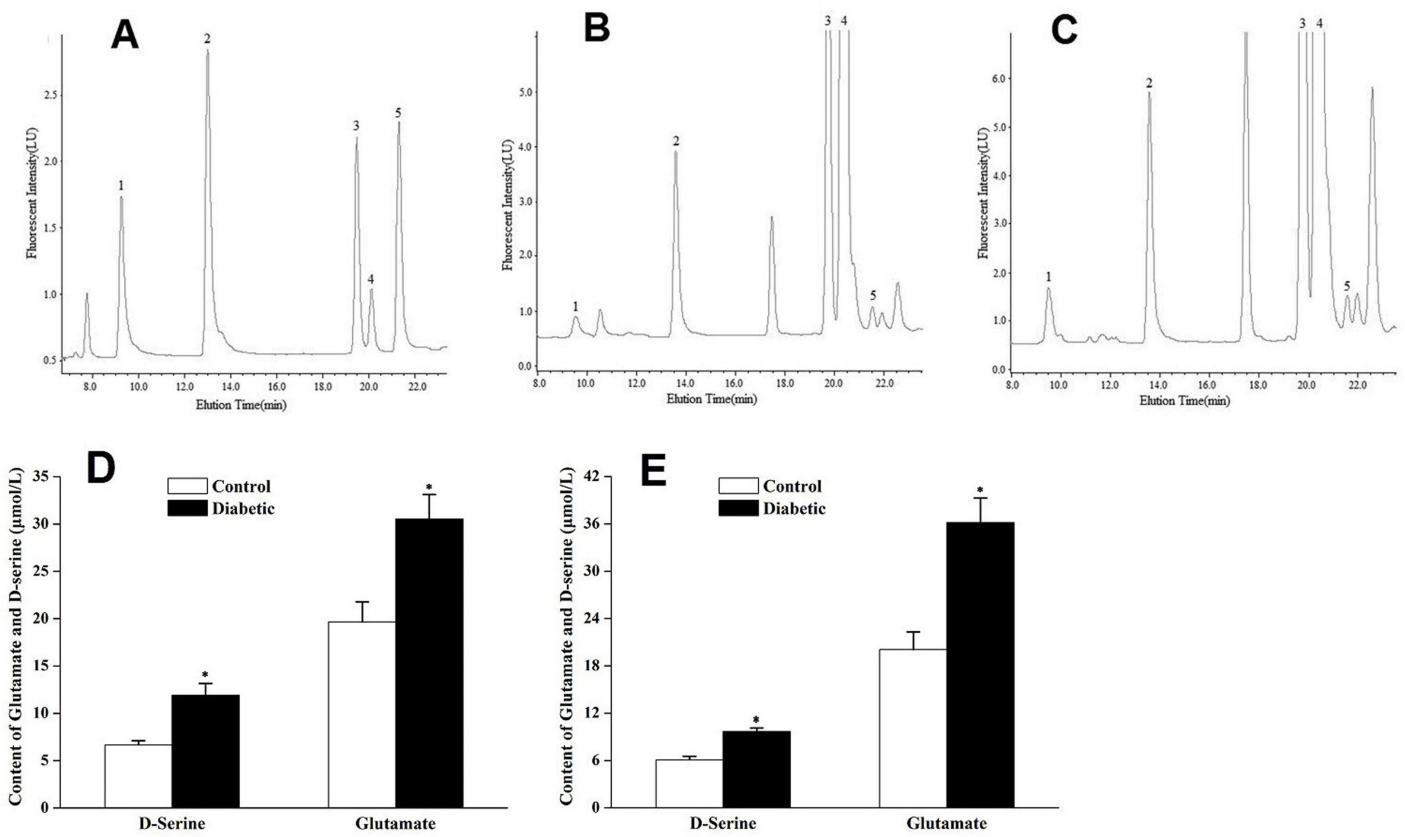
**Increased D-serine and glutamate in aqueous humor of DR rats determined by HPLC**. **A: **Amino acid standards were separated by reverse-phase HPLC; 1: L-Asp, T_R _= 9.243 min; 2: L-Glu, T_R _= 12.995 min; 3: L-Ser, T_R _= 19.472 min; 4: L-Gln, T_R _= 20.108 min; 5: D-Ser, T_R _= 21.302 min. **B, C: **Aqueous humor samples from control rats (**B**) or from DR rats (**C**) at 3 months after onset of diabetes. **D: **Quantification of glutamate and D-serine in aqueous humor from DR and control rats at 3 months after onset of diabetes. **E: **Quantification of glutamate and D-serine in aqueous humor from DR and control rats at 5 months after onset of diabetes. The results shown are mean ± SEM from triplicate experiments (*P < 0.05 vs. control).

Because levels of SR were found to be elevated in retinas, we next examined whether this translated into an increase in D-serine levels. Levels of D-serine showed a trend toward somewhat higher levels in diabetic rat retina 3 months after STZ, but there was not a significant difference at either time point. The RGC population may be vulnerable to excitotoxins that exist in ocular humor, levels of which would not be detected in assays of neural retina homogenates. We tested D-serine and glutamate in aqueous humor and found significant elevations of both of these excitatory amino acids in DR rats (Figure 4). We also attempted to assay D-serine in vitreous humor but the lens of the DR rats adhered to the retina so that the vitreous humor of DR rats was not easily isolated.

### Increased phospho-JNK in retinas of DR

**Figure 5:**
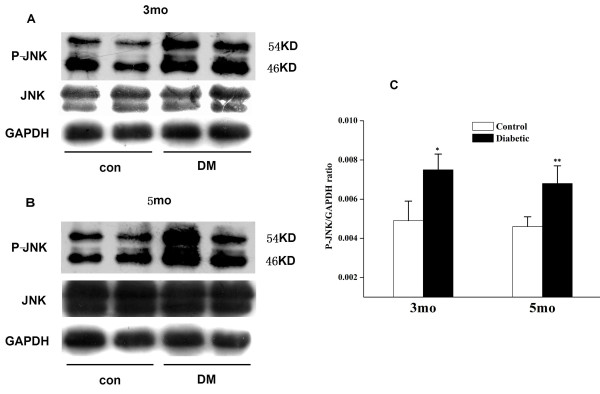
**Increased phospho-JNK in retinal homogenates from DR rats**. Retinal homogenates from control and DR rats at 3 (**A**) or 5 (**B**) months after onset of diabetes were subjected to immunoblotting for phospho-JNK, with 50 μg total protein loaded in each lane. Compared to control, DR retinal homogenates showed increased phospho-JNK (54 and 46 kDa) at both 3 and 5 months after onset of diabetes; no increase in total JNK was detected. (**C**) Densitometric scans indicate that the ratio between SR and GAPDH in DR rats is significantly higher than control (*P < 0.05, **P < 0.05). The results shown are typical of duplicate experiments.

Previous reports indicate that the JNK pathway is activated in diabetes mellitus [35,36], and JNK activity is increased in DR [37]. We have demonstrated that inflammation increases SR expression in microglial cells via activation of the JNK pathway, which culminates in binding of a c-Fos/JunB transcription-factor complex to an AP-1 site in the SR intron 1c [31]. Therefore, we tested whether JNK contributes to increased SR expression in DR by assaying relative levels of phospho-JNK (54 and 46 kDa) in retinal homogenates. Compared with control, increased phospho-JNK was detected in DR homogenates at 3 or 5 months after onset of diabetes (Figure 5). By contrast, no increase in total JNK was detected, suggesting activation of extant kinase.

## Discussion

Our results indicate that SR is elevated in retina and D-serine is increased in aqueous humor in the STZ-induced model of DR. The increased SR expression in retina may result from activation of the JNK pathway in DR. To our knowledge, this is the first report of an increase in the levels of SR and D-serine in DR. We also found that glutamate levels in DR retina are ~1.5-fold higher than control, consistent with a report by Lieth *et al*. that glutamate is ~1.6-fold higher in DR retina [15].

We found that levels of total D-serine in retina are ~100-fold lower than those of glutamate (not shown); but this is consistent with their relative total concentrations in other neural tissues, reflecting the distinctions in compartmentalization and metabolic roles for these two amino acids. There were no significant differences in retinal D-serine between DR rats and controls, which may result from spillover of excess retinal D-serine into the ocular humors. Compared to those in adult retina, levels of D-serine were easily detected by reverse-phase HPLC in aqueous humor of adult rats, where D-serine levels were only one fifth those of glutamate. We also noticed that SR or D-serine were higher at 3 months after onset of diabetes than at 5 months after onset of diabetes. Possible explanations include the previously reported decline in SR expression with aging [38].

Increased SR expression in retina was positively correlated with JNK pathway activation, indicated by increased levels of phospho-JNK. Currently, we do not know which isoforms of JNK regulate SR expression in DR retina. JNK1 and JNK2 are found in all cells and tissues and their functions are redundant, and JNK3 is mostly localized in brain [39]. Thus, it seems likely that JNK1 or JNK2 is responsible for regulating SR expression by inflammation in DR retina. We previously demonstrated that downstream of JNK, a c-Fos/JunB complex is responsible for regulating SR expression by inflammatory stimuli in microglia [31]. In DR retina, we did detect increased phospho-JNK but not increased phospho-c-Jun or JunD. Potential changes in phospho-JunB in DR retina will be investigated in future studies.

In our study, increased SR was found primarily in INL. Judging from morphology, these are glial cells containing strong SR staining. These may include Müller cells, astrocytes, or other glial cells in retina expressing SR [20,38,40]. Retinal homogenates also contained an SR dimer resistent to the denaturation conditions of SDS-PAGE, as we previously documented for microglia [16], though in much smaller amounts than monomers (not shown).

Previous results have indicated that intravitreal injection of D-serine or glycine can enhance NMDA toxicity towards RGCs, whereas blocking the glycine_B _binding site with 5,7-dichlorokynurenic acid (DCKA) or blocking glycine transport reduces toxicity [41]. Our results indicate increased levels of glutamate and D-serine in aqueous humor of DR rats and increased glutamate in retina as well; the increased glutamate in DR is consistent with another prior report [15]. Taken together, our data indicate that increased D-serine in the enclosed environment of eyes may exacerbate glutamate toxicity towards RGCs in DR.

Our results also indicated that vWF staining does not overlap with TUNEL staining (not shown), which suggests that endothelial cell death is not substantial at 3 or 5 months post-STZ injection. Previous reports have indicated that breakdown of the blood-retinal barrier (BRB) is limited, if not altogether absent, at early stages of STZ-induced DR [42,43]. These results suggest that leakage of leukocytes or their products due to BRB breakdown do not make a substantial contribution to RGC death. Nevertheless, leukocytes can extravasate through endothelial barriers, even in healthy vessels [44]. Once there, they may become activated by AGEs, molecules which could also contribute directly to neurodegenerative events [45,46]. In addition, blood-borne leukocytes or activation of resident glia can compromise neuronal function and viability via oxidative stresses, release of proteases, and the pathological production of prostanoids [47]. However, our work demonstrates that elevations in glutamate and D-serine may contribute to these inflammatory sequelae occurring in DR.

## Abbreviations

SR: serine racemase; STZ: streptozotocin; DR: diabetic retinopathy; HPLC: high-pressure liquid chromatography.

## Competing interests

The authors declare that they have no competing interests.

## Authors' contributions

Author 1 (H-YJ) established the DR rat model and performed western blotting, immunofluorescence, H&E staining, TUNEL assays, and HPLC measurements. Author 2 (J-XF) contributed to western blotting. Author 3 (BW) helped establish the DR rat model. Author 4 (G-BY) performed western blotting for phospho-JNK and phospho-c-Jun. Author 5 (LS) performed immunofluorescence for vWF. Author 6 (JQ) provided expert opinions on the project. Author 7 (SWB) provided expert opinions on the project and contributed to writing of the manuscript, as well. Author 8 (S-ZW) conceived of this study, participated in its design and coordination, and wrote the manuscript. All authors read and approved the final manuscript.
